# Field Assessment of Naled and Its Primary Degradation Product (Dichlorvos) in Aquatic Ecosystems Following Aerial Ultra-low Volume Application for Mosquito Control

**DOI:** 10.1007/s00244-023-00981-8

**Published:** 2023-03-13

**Authors:** Cassandra D. Smith, Michelle L. Hladik, Kathryn M. Kuivila, Ian R. Waite

**Affiliations:** 1 USGS Oregon Water Science Center, Portland, OR USA; 2 USGS California Water Science Center, Sacramento, CA USA; 3 USGS Scientist Emeritus, Portland, USA

## Abstract

Naled, an organophosphate insecticide, is applied aerially at ultra-low volumes over aquatic ecosystems near Sacramento, California, USA, during summer months for mosquito control. Two ecosystem types (rice fields and a flowing canal) were sampled in 2020 and 2021. Naled and its primary degradation product (dichlorvos) were measured in water, biofilm, grazer macroinvertebrates, and omnivore/predator macroinvertebrates (predominantly crayfish). Maximum naled and dichlorvos concentrations detected in water samples one day after naled application were 287.3 and 5647.5 ng/L, respectively, which were above the U.S. Environmental Protection Agency’s aquatic life benchmarks for invertebrates. Neither compound was detected in water more than one day after the application. Dichlorvos, but not naled, was detected in composite crayfish samples up to 10 days after the last aerial application. Detections in water from the canal showed that the compounds were transported downstream of the target application area. Factors such as vector control flight paths, dilution, and transport through air and water likely affected concentrations of naled and dichlorvos in water and organisms from these aquatic ecosystems.

**Graphical Abstract:**

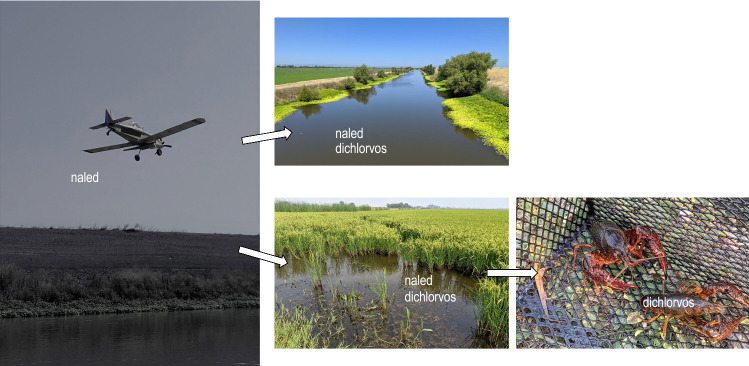

Organophosphate insecticides are often chosen for vector control because of their acute toxicity and short half-lives in the environment (Desouky et al. 2013). During the Zika virus outbreak in the United States in 2016, vector control agencies began to apply naled (an organophosphate insecticide) more frequently, and over areas that were not previously sprayed with pesticides (Jones et al. 2020; McAllister et al. 2020). The increased use of naled highlighted the need for more information regarding the fate and transport of this insecticide in various environmental conditions and aquatic ecosystems.

Naled is often applied aerially at ultra-low volumes (ULVs), which can reduce application costs and optimize droplet size for adult mosquito control (Mount et al. 1996). However, naled is applied directly over aquatic ecosystems, and spray drift can introduce the pesticide to non-target ecosystems. Past studies have shown that measurable concentrations of naled are detected in the air (Schleier and Peterson 2010b; Zhong et al. 2010), water (Jones et al. 2010; Phillips et al. 2014), and on surfaces (Pierce 2008) following ULV applications for adult mosquito control. After an aerial application, naled was detected on residue samplers 19 km from the target spray area (Zhong et al. 2010). Following a series of naled spray events, Phillips et al. (2014) collected water samples from various environmental settings, and two samples exceeded the California National Pollutant Discharge Elimination System (NPDES) permit trigger value, or regulatory threshold, for naled.

Naled primarily degrades through photolysis and hydrolysis (Tietze et al. 1996). Therefore, ultraviolet radiation and humidity affect the degradation of naled in air and on terrestrial surfaces, and the half-life on surfaces can range from 1.4 to 8 h (Tietze et al. 1996). The degradation of naled in freshwater is affected by light, organic carbon, and temperature, and the half-life at 23 °C is approximately 15 min when exposed to light and 20 h in the dark (Jones et al. 2020). Naled is used to directly kill nuisance organisms, but the organophosphate insecticide is non-specific and can affect non-target species (Schleier and Peterson 2010a). While naled degrades quickly in most field conditions, the breakdown product dichlorvos (2,2-dichlorovinyl dimethyl phosphate) is also an organophosphate insecticide and a possible human carcinogen (Cox 2002). In dark conditions at 23 °C, the half-life of dichlorvos is about 7 days, but light conditions can result in rapid degradation (half-life < 67 min; Jones et al. 2020). Following naled applications, the toxicity of water samples to the non-target freshwater invertebrate *Ceriodaphnia dubia* was mainly attributed to dichlorvos concentrations in the water (Phillips et al. 2014).

Naled has a low potential to accumulate in animal tissues (Stavola 2004); however, dichlorvos has been shown to accumulate in damselfly larvae (Van Praet et al. 2014), mussels (Bashnin et al. 2019; Wille et al. 2011), and fish (Barbieri et al. 2019; Brodeur et al. 2017). The effects of dichlorvos exposure may be sublethal, and higher concentrations have been correlated with decreased body mass in damselfly larvae (Van Praet et al. 2014). Organophosphate insecticides also can accumulate in crayfish, which are an important food source for humans in many regions of the world (Desouky et al. 2013; Xiong et al. 2020). Studies have shown that other organophosphate insecticides (fenitrothion and ethion) and their degradation products remained in crayfish abdominal muscles 7 days after exposure in laboratory experiments (Desouky et al. 2013; Escartín and Porte 1996).

In areas of the United States such as California, vector control operations are tightly regulated, and applications of naled often are alternated with applications of pyrethrins to prevent mosquito resistance (McGregor and Connelly 2021). Naled is registered and regulated under the Federal Insecticide, Fungicide, and Rodenticide Act (US EPA 2002). In addition, the California State Water Resources Control Board regulates vector control pesticide applications with a NPDES permit (2016-0039-DWQ; CA Water Boards 2022). The NPDES permit regulates the discharge of the resulting residues of toxicological concern from the application. Naled is regulated with a permit trigger value of 14 ng/L (CA Water Boards 2022); since dichlorvos is a degradation product, it is currently not regulated with an NPDES permit. In addition to California’s NPDES trigger value, aquatic life toxicity benchmarks are published by the U.S. Environmental Protection Agency (US EPA) Office of Pesticide Programs. Acute toxicity benchmarks for invertebrates for naled and dichlorvos are 57.5 and 33.4 ng/L, respectively; chronic benchmarks for invertebrates for naled and dichlorvos are 10 and 5.8 ng/L, respectively (US EPA 2022b).

The fate and transport of naled and dichlorvos following a ULV application are dependent on the weather (Schleier and Peterson 2010b), time of ULV application (Mount et al. 1996), and site-specific characteristics such as water movement (Phillips et al. 2014; Pierce et al. 2005). Since naled is applied during evening hours (when photolysis is minimal) in the central valley of California, both the parent compound and degradation products may persist and could potentially be transported away from the application area (Jones et al. 2020; Pierce et al. 2005; Tietze et al. 1996). In flowing waters, exposure to aquatic organisms may be ephemeral (Stavola 2004), and mixing may slightly increase hydrolysis rates (Noblet et al. 1996). By comparison, shallow, lentic systems may concentrate parent compounds and degradation products because of less dilution. Following ULV applications, exposure to aquatic organisms may vary based on the volume of compounds reaching the water body, surrounding concentrations in the water body, and their food sources. Aquatic organisms within grazing and scraping functional feeding groups may uptake dichlorvos through biofilms. We hypothesized that concentrations of naled and dichlorvos in aquatic environments following ULV applications would be higher in lentic than in lotic environments, for reasons discussed above, and that dichlorvos would be detected more frequently than naled in biota because of the low potential for naled to accumulate in animal tissues.

## Methods

Naled typically is applied aerially around Sacramento, California, USA, from late July through mid-October each year, and the frequency of application depends on mosquito abundance and disease presence. Dibrom^®^ (active ingredient: naled [87.4%]) is applied using a fixed-wing aircraft at a rate of 0.07 kg of active ingredient per hectare (0.06 pounds of active ingredient per acre). Naled and dichlorvos also can originate from sources other than adult mosquito control operations. For example, naled is used on agricultural crops (US EPA 2002), and trichlorfon degrades into dichlorvos (Li et al. 2011). However, trichlorfon is not registered for use in California, and the California Pesticide Use Reporting database confirmed that detections during this study were a result of the ULV naled applications for vector control (CA DPR 2022).

### Study Areas

**Fig. 1:**
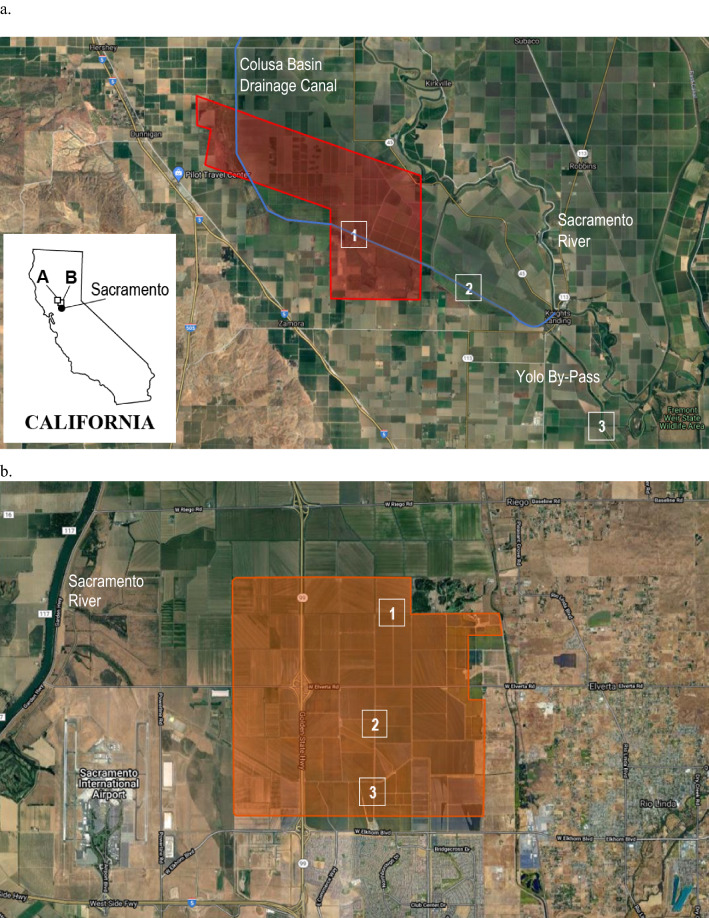
Typical flight areas (shown in red and orange) to apply ultra-low volume insecticides for adult mosquito control over (**a**) the Colusa Basin Drainage Canal and (**b**) rice fields near Sacramento, California. The Colusa Basin Drainage Canal (shown in blue) drains into either the Sacramento River or the Yolo By-Pass. Spray areas varied based on weather, nuisance mosquito abundance, and wildfire smoke. Background images from Google, and spray areas provided by the Sacramento-Yolo Vector Control District. Numbers indicate sampling locations

The Colusa Basin Drainage Canal (CBDC; also referred to as the Colusa Basin Drain) is a conduit that drains agricultural fields north of Sacramento, California, and discharges into the Yolo By-Pass waterway or the Sacramento River at Knights Landing. The canal is an engineered ditch that ranges from approximately 30–85 m wide and 1.5–3 m deep. Three sampling locations (CBDC1, CBDC2, and CBDC3; Fig. 1a) from upstream to downstream along the canal were sampled to assess whether naled and dichlorvos were transported downstream.

Vector control flight paths varied based on nuisance mosquito abundance and weather. During summers 2020 and 2021, flight paths around the Colusa Basin Drainage Canal usually applied naled upstream of CBDC1 and over the fields north of the canal (Fig. 1a). Naled was applied directly over CBDC1 during approximately half the applications. CBDC2 was 6.8 km downstream of CBDC1, and CBDC3 was 10.8 km downstream of CBDC2 and located along the Yolo By-Pass; neither downstream site was directly sprayed during any of the aerial applications for vector control. It is not known if spray drift affected CBDC2; the eastern-most edge of the Colusa Basin Drainage Canal flight path was approximately 3.5 km from CBDC2. During the two target applications at the Colusa Basin Drainage Canal in 2020 and 2021, average wind speeds were 10.3 kph, and average wind directions were 157 and 150 degrees, respectively (station name: Verona; CIMIS 2022).

Rice fields north of Sacramento were selected as lentic systems resembling wetlands. These fields are terraced and interspersed with ditches; therefore, water is often circulating through the fields and ditches but can be relatively stagnant in the corners. Three sampling locations (Rice1, Rice2, and Rice3; Fig. 1b) were selected in a north–south transect, and all three sites were directly sprayed with each aerial naled application. During the 2020 target application over the rice fields, average wind speed was 8.5 kph and average wind direction was 155 degrees (station name: Verona; CIMIS 2022). Average wind speed was 1.9 kph, and average wind direction was 226 degrees during the 2021 target application (CIMIS 2022). Sampling locations in the Colusa Basin Drainage Canal and rice fields were chosen to assess concentrations of naled and dichlorvos (1) in water and aquatic trophic levels, (2) among lentic and lotic systems, and (3) over time following ULV applications.

### Sample Collection

**Fig. 2:**
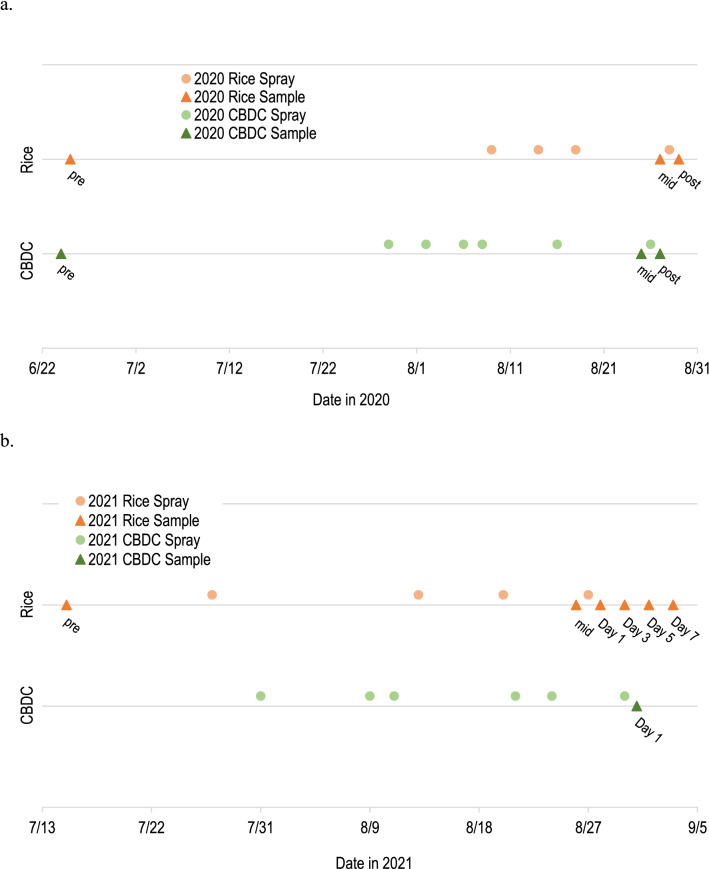
Dates of aerial applications of Dibrom^®^ (active ingredient: naled [87.4%]) and sample collection from the Colusa Basin Drainage Canal and rice fields near Sacramento, California, in **a** 2020 and **b** 2021. Samples were collected pre-season, mid-season, and post-application in 2020. Samples were collected pre-season, mid-season, and on Days 1, 3, 5, and 7 in 2021

In 2020, samples of water, biofilm, and macroinvertebrates were collected from each location on three dates: (1) prior to the insecticide application season (June; pre-season), (2) after multiple naled applications and immediately before the target application (mid-season), and (3) the day after the target naled application (post-application; Fig. 2a). Water samples were collected as grab samples in baked 1-L amber glass bottles. At each site, the glass bottle was submerged and held under the surface until it filled to the top to reduce headspace and minimize volatilization. Water samples were immediately placed on ice and remained chilled and in the dark until analysis to reduce photolysis and other abiotic or biotic processes.

Biofilm was scraped from aquatic vegetation and other surfaces (including submerged garbage in the canal) with a stainless-steel spatula into a baked glass jar until a sufficient mass was obtained for analysis. Macroinvertebrates were collected from vegetation and substrate using nets and were placed into larval trays. Macroinvertebrates were sorted between grazers/detritivores and omnivores/predators from the trays and placed into screw top conical tubes. Grazer macroinvertebrates consisted mainly of crustaceans and gastropods. Omnivore/predator macroinvertebrates consisted of dragonfly and damselfly nymphs, diving beetle larvae, and crayfish. Samples were immediately placed on ice in a cooler, and equipment was cleaned with methanol between sites.

Water samples were collected in the middle of the channel from bridges from a depth of approximately 0.5 m at CBDC2 and CBDC3; biofilm and macroinvertebrate samples were collected by wading. A thick mat of macrophytes (*Ludwigia* spp.) on the left bank and extending toward the middle of the canal was the access and sampling location at CBDC1. In the rice fields, samples were collected from the corners of the fields and, if needed, in the adjacent ditches to collect sufficient material for analysis.

Sample collection in 2021 was focused on water and crayfish in the rice fields. Water and crayfish were collected (1) prior to the insecticide application season (July; pre-season), (2) after multiple naled applications and immediately before a target application (mid-season), and (3) days 1, 3, 5, and 7 following a naled application (Fig. 2b). Crayfish traps were used to collect three composites of three crayfish (9 total) from each site on each day. Traps were baited with canned cat food that was partially opened to prevent ingestion. One crayfish composite and water sample were collected from CBDC2 the day after a naled application (Day 1).

Samples collected prior to the insecticide application season (pre-season) indicated whether residuals from the prior application season persisted. Mid-season samples in both years were used to determine if the target compounds were persisting between applications. Post-application (or Days 1–7) samples were used to assess the direct effect of an aerial application.

### Laboratory Analysis

Samples were analyzed for naled and dichlorvos concentrations at the U.S. Geological Survey (USGS) Organic Chemistry Research Laboratory in Sacramento, California. Prior to extraction of all sample matrices, *d*_4_-imidacloprid was added as a recovery surrogate to assess the performance of the solid phase extraction procedure. Water-soluble *d*_4_-imidacloprid is used as a surrogate in the multi-residue pesticide method (Gross et al. 2021), and laboratory test studies have shown that the surrogates were similar in recovery to naled and dichlorvos. Water samples (1 L) were refrigerated, filtered using 0.7-μm glass-fiber filters, and extracted within 24 h of collection using solid-phase extraction (Waters Oasis HLB; 6 mL, 500 mg); final extracts were 0.2 mL in acetonitrile (details in Gross et al. 2021). Biota samples (biofilm and macroinvertebrates) were frozen until analysis and homogenized prior to extraction. Crayfish were mechanically cut with pre-cleaned scissors to achieve homogenization prior to extraction while minimizing the loss or potential degradation of the target compounds. Biota sample masses ranged from 0.1 to 92.6 g. Biota samples were extracted using 50:50 acetone:dichloromethane (enough to cover the sample) and were sonicated for 10 min. The solvent was decanted through sodium sulfate, and the sample was extracted a second time; the two solvent extractions were combined, evaporated, and exchanged into acetonitrile (final volume 0.2 mL). All samples were quantitated using liquid chromatography-tandem mass spectrometry (Gross et al. 2021). The estimated limits of detection in water were 1 ng/L for dichlorvos and 10 ng/L for naled. The estimated limits of detection for the biota samples varied by sample mass; limits of detection ranged from < 0.1 to 17.0 ng/g for dichlorvos and 0.1 to 169.5 ng/g for naled (92.6–0.1 g samples, respectively).

Recovery of *d*_*4*_-imidacloprid for all water and biota samples was within the range of 70–128%. Neither naled nor dichlorvos was detected in the blank water sample. For the biota samples, procedural laboratory blanks were run using baked sand with no detects of naled or dichlorvos. Due to the limited amount of biota sample, only one matrix spike in the laboratory was done for a grazer macroinvertebrate, and compound recoveries were 104 and 72% for dichlorvos and naled, respectively. The USGS Organic Chemistry Research Laboratory met all instrumentation parameter checks (including calibration curves, blanks, and check standards) during 2020 and 2021, and a review of the quality assurance and quality control data did not affect interpretation.

## Results

Thirty-three water samples were collected over the two-year study, and 24% had detections of naled and/or dichlorvos. The water samples with detections were all collected the day after the target ULV naled applications. All other water samples collected during the study (pre-season, mid-season, Day 3, Day 5, and Day 7) were below the limit of detections for the compounds.

**Fig. 3:**
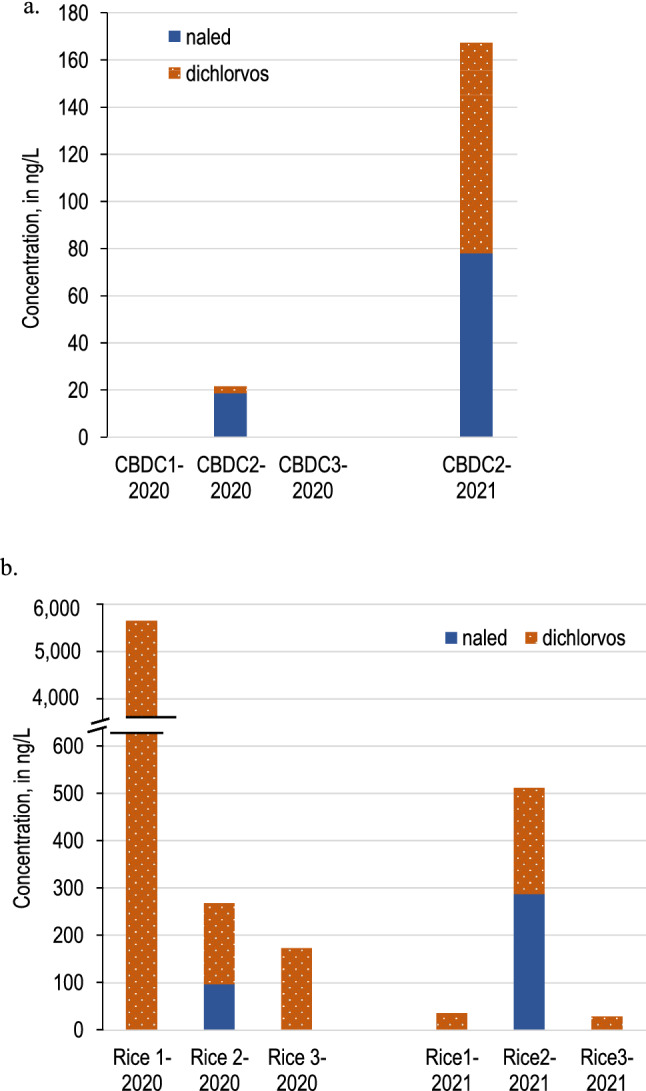
Concentrations (ng/L) of naled and dichlorvos detected in water samples from a) a drainage canal and b) rice fields near Sacramento, California, collected the day after (post-application in 2020; Day 1 in 2021) an ultra-low volume naled application in 2020 and 2021. One water sample was collected from each site. Naled and dichlorvos concentrations are stacked. Naled and dichlorvos concentrations in water samples collected on all other days during the study were below the limit of detection

Both naled and dichlorvos were detected in water at CBDC2 following an aerial application (post-application) in 2020, and the naled concentration of 18.6 ng/L was greater than the NPDES level of 14 ng/L (Fig. 3a). The aerial extent of the insecticide application in the evening of August 26, 2020, was reduced owing to visibility concerns from wildfire smoke. On that date, the flight path only encompassed the agricultural fields north of the canal, and naled was not sprayed directly over the canal. Similarly, naled and dichlorvos were detected on Day 1 in 2021 at CBDC2 (Fig. 3a): Naled was 77.9 ng/L (approximately 5.6 times greater than the NPDES trigger value) and dichlorvos was approximately 2.7 times greater than the acute toxicity benchmark for invertebrates at 89.3 ng/L. The aerial ULV naled application that occurred the evening before sample collection in 2021 at the Colusa Basin Drainage Canal was applied directly to the canal and fields adjacent to CBDC1, as shown in Fig. 1a.

Following the target insecticide application over the rice fields in 2020, there was substantial spatial variation in concentrations within the rice field complex (Fig. 3b). Naled was 96.8 ng/L in water at Rice2. Dichlorvos was detected at orders of magnitude greater than the aquatic life benchmarks for invertebrates in water at all 3 rice sites on the post-application day. Dichlorvos concentrations at Rice2 and Rice3 were similar and averaged 172.2 ng/L, while the concentration at Rice1 was approximately 33 times higher at 5647.5 ng/L.

In 2021, dichlorvos was detected at all 3 rice sites in lentic water on Day 1, and naled was detected at Rice2 on Day 1 (Fig. 3b). At Rice2, both naled and dichlorvos concentrations were > 200 ng/L, which was substantially greater than the acute toxicity benchmarks and the naled NPDES trigger value. Concentrations of dichlorvos on Day 1 at Rice1 and Rice3 were similar with an average of 33.1 ng/L. Neither compound was detected in flowing water at Rice3 on Day 1. Naled and dichlorvos were not detected in water on Days 3, 5, or 7 at any location.

Neither naled nor dichlorvos was detected in biofilm or grazer macroinvertebrate samples from any canal or rice sites (Table 1). The average masses of the biofilm samples and the grazer macroinvertebrate samples were both 0.3 g (g) of wet weight, and low sample masses may be the reason that naled and dichlorvos were not detected. Omnivore macroinvertebrate sample masses in 2020 ranged from 0.1 to 40.8 g of wet weight; the only samples with dichlorvos detections had > 14 g and each contained a crayfish. All 5 dichlorvos concentrations detected in omnivore macroinvertebrate samples in 2020 were 1 to 2 orders of magnitude higher than the associated limit of detection (Table 1; Smith et al. 2022).

**Table 1:** Concentrations of naled and dichlorvos in biofilm and invertebrate samples collected from a canal and rice fields near Sacramento, California

Day	Date (m/dd/yyyy)	Site	Biofilm		Grazer invertebrate		Omnivore invertebrate	
			Naled	Dichlorvos	Naled	Dichlorvos	Naled	Dichlorvos
Pre-season	6/24/2020	CBDC1	nd	nd	nd	nd	nd	nd
		CBDC2	nd	nd	nd	nd	nd	nd
		CBDC3	–	–	–	–	–	–
Mid-season	8/26/2020	CBDC1	nd	nd	nd	nd	nd	nd
		CBDC2	nd	nd	nd	nd	nd	nd
		CBDC3	–	–	–	–	–	–
Post-application	8/27/2020	CBDC1	nd	nd	nd	nd	nd	nd
		CBDC2	nd	nd	nd	nd	nd	nd
		CBDC3	–	–	–	–	–	–
Pre-season	6/25/2020	Rice1	nd	nd	nd	nd	nd	nd
		Rice2	nd	nd	nd	nd	–	–
		Rice3	nd	nd	nd	nd	nd	nd
Mid-season	8/28/2020	Rice1	nd	nd	nd	nd	nd	0.6
		Rice2	nd	nd	nd	nd	nd	2.7
		Rice3	nd	nd	nd	nd	nd	3.8
Post-application	8/29/2020	Rice1	nd	nd	nd	nd	nd	0.9
		Rice2	nd	nd	nd	nd	nd	nd
		Rice3	nd	nd	nd	nd	nd	0.6

Concentrations in biofilm and invertebrates are in ng/g of wet weight. Abbreviations: *CBDC* Colusa Basin Drainage Canal; *nd* analyte not detected; – sample not analyzed

Naled and dichlorvos were not detected in any pre-season samples (June 2020 and July 2021) of water, biofilm, or macroinvertebrates (Tables 1 and 2; limits of detection and water data available in Smith et al. 2022). In 2020, three naled applications had occurred over the rice fields and five applications had occurred around the canal before the mid-season samples were collected. Naled and dichlorvos were not detected in mid-season water samples in 2020 from the rice fields or the canal. Dichlorvos was detected in mid-season omnivore macroinvertebrate samples from all three rice sites (8/28/2020; Table 1). In this case, the previous naled application had occurred 10 days before the mid-season omnivore samples were collected (Fig. 2a). Dichlorvos was detected in omnivore samples at two rice sites on the post-application day (8/29/2020; Table 1) following the aerial application; concentrations varied but were generally lower than the concentrations detected in the mid-season samples from the previous day.

**Table 2:** Concentrations (ng/g of wet weight) of naled and dichlorvos in crayfish composite samples collected from rice fields and a canal north of Sacramento, California, 2021

Day	Date (m/dd/yyyy)	Site	Crayfish composite 1		Crayfish composite 2		Crayfish composite 3	
			Naled	Dichlorvos	Naled	Dichlorvos	Naled	Dichlorvos
Pre-season	7/15/2021	Rice3	nd	nd	nd	nd	nd	nd
Mid-season	8/27/2021	Rice1	nd	nd	–	–	–	–
		Rice2	nd	nd	–	–	–	–
		Rice3	nd	nd	nd	nd	–	–
1	8/28/2021	Rice1	nd	0.33	–	–	–	–
		Rice2	nd	nd	nd	0.07	nd	nd
		Rice3	nd	nd	nd	nd	nd	nd
3	8/30/2021	Rice1	nd	nd	nd	nd	nd	nd
		Rice2	nd	nd	nd	0.06	nd	nd
		Rice3	nd	nd	nd	0.07	nd	nd
5	9/1/2021	Rice1	nd	nd	nd	nd	nd	nd
		Rice2	nd	nd	nd	nd	nd	nd
		Rice3	nd	nd	nd	nd	nd	nd
7	9/3/2021	Rice1	nd	nd	nd	nd	nd	nd
		Rice2	nd	nd	nd	nd	nd	nd
		Rice3	nd	nd	nd	nd	nd	nd
1	8/31/2021	CBDC2	nd	nd	–	–	–	–

Abbreviations: *CBDC* Colusa Basin Drainage Canal, *nd* analyte not detected, – sample not collected. An ultra-low volume aerial application of naled occurred on the evenings of 8/27/2021 (Rice) and 8/30/2021 (CBDC)

Low concentrations of dichlorvos ranging from 0.06 to 0.33 ng/g were detected in crayfish from two rice sites on Days 1 and 3 in 2021; naled was not detected in any crayfish (Table 2). A single opportunistic adult dragonfly sample was collected at Rice3 on Day 1 in 2021. Naled was not detected in the adult dragonfly sample, but dichlorvos was 2.68 ng/g.

## Discussion

In this study, high concentrations of naled and/or dichlorvos were detected in water from most canal and rice sites the mornings after ULV naled applications, but both naled and dichlorvos degraded to concentrations below detection levels in water before Day 3. Concentrations generally were higher in lentic compared to lotic waters; summed naled and dichlorvos concentrations that were detected ranged from 29.6 to 5647.5 ng/L in the rice fields and < 1 to 167.2 ng/L in the canal sites. Naled and dichlorvos were not detected in biofilm or grazer macroinvertebrate samples from this study, but dichlorvos was detected in crayfish up to 10 days after an aerial application. The presence of dichlorvos in crayfish when the analyte was not detected in the co-located water sample (mid-season in 2020 and Day 3 in 2021) indicates that dichlorvos may accumulate in crayfish following ULV naled applications for vector control.

Results from the canal sites show that naled was transported outside of the spray area in both 2020 and 2021. The concentrations of the compounds in water were likely affected by the area sprayed, because higher concentrations were detected in 2021 when the spray area was larger and included portions of the canal. Naled and dichlorvos have been detected in water samples in other years along this canal outside of the spray area (US Geological Survey 2022). This study did not determine whether the presence of naled and dichlorvos in water at CBDC2 was a result of spray drift or transport in water, or how many days after the application the detections persisted in the canal. However, based on the timing of naled applications (between 7 pm and 12 am) and sample collection the following mornings, naled and dichlorvos were detected in the aquatic environment at least 9 h after application. The presence of the compounds and the concentrations detected (exceeding the NPDES permit trigger value for naled in 2020 and acute invertebrate toxicity benchmarks for both naled and dichlorvos in 2021) may be a result of evening, as opposed to morning, applications. Considering that naled and dichlorvos were not detected at the downstream site (CBDC3), the compounds may be degraded or diluted within a few kilometers, and flowing water may facilitate degradation, dissipation, or transport. Future efforts, such as deploying residue samplers in the air, could help characterize the transport of naled and dichlorvos into the canal and the spatial and temporal persistence.

While naled often breaks down quickly to dichlorvos in field settings, high concentrations of dichlorvos are detected in water in some aquatic systems following ULV applications. Phillips et al. (2014) found zero percent survival of *Ceriodaphnia dubia* when dichlorvos concentrations were > 100 ng/L in water. Dichlorvos concentrations detected in this study exceeded 100 ng/L multiple times at lentic rice sites; dozens of dead snails and crayfish were observed following applications, but it was not clear if mortality was a result of the naled applications. Both naled and dichlorvos degraded quickly in the aquatic environments, as neither compound was detected in water samples after Day 1. Currently, dichlorvos is not included in the California NPDES permit system, but detection in both water and aquatic organisms in this study supports the importance of monitoring dichlorvos in addition to naled in receiving waters.

Results about the fate and transport of pesticides in rice fields may be transferrable to other wetland-analogous systems. Rice fields around Sacramento support a wide variety of birds including ibis, herons, egrets, and kites. Naled is toxic to birds (US EPA 2002), and birds may be repeatedly exposed during an application season. As water levels decrease in the rice fields and ditches, many birds prey on stranded crayfish. Based on the toxicity of dichlorvos and the ability for it to persist in crayfish, assessing sublethal effects on local birds may be warranted. In this study, dichlorvos also was detected in a single, opportunistic sample of adult dragonflies captured in the rice fields on Day 1 (Rice3; Smith et al. 2022). These terrestrial insects may transport dichlorvos to other areas and expose additional predators.

People catch and consume sometimes large quantities of crayfish from the ditches and rice fields in the Sacramento area. In addition to organophosphate insecticides detected in this study, other studies have shown that crayfish accumulate heavy metals and cyanotoxins (Liras et al. 1998; Xiong et al. 2020), indicating that crayfish in agricultural areas may contain multiple contaminants of concern. Unintentional chemical mixtures in the environment change in composition and concentration over time and, therefore, determining human health risks for specific mixtures is largely impractical (Kienzler et al. 2016). Considering that dichlorvos is a possible human carcinogen (Cox 2002), determining whether dichlorvos remains in the edible tissues of prepared crayfish would help assess whether this exposure pathway may affect human health.

This field study was designed to assess exposure of resident biota to pesticides used for vector control operations during typical environmental conditions. However, collecting enough material (such as biofilm and macroinvertebrates) was challenging and resulted in widely varying sample masses, which can affect analyte detection. In addition, the inconsistency of naled and dichlorvos concentrations over time in environmental samples may be affected by variability in individual crayfish. Future studies could deploy caged macroinvertebrates or materials (such as unglazed tiles) where biofilm can adhere in order to standardize sample sizes, quantify pesticides per area, and characterize effects. Pesticides can be transformed by biofilms (Sánchez-Pérez et al. 2013) and can affect biofilm community structure (Tiam et al. 2014), but it is currently unclear how naled transformation and dichlorvos degradation are affected by biofilms.

Wildfire smoke was present and dense at times during this study. In addition to factors such as wind speeds (Tiryaki and Temur 2010), air and water temperatures, and humidity, particulate matter in the air may affect the degradation of pesticides in aquatic environments. Wildfires have been increasing in the western US over the past few decades (Abatzoglou and Williams 2016), and particulate matter in the air during wildfires can increase by more than 100 μg m^−3^ (Wu et al. 2006). Elevated concentrations of particulate matter can decrease air and water temperatures, attenuate solar radiation, and reduce photolysis (Da Silva et al. 2012; David et al. 2018; Williamson et al. 2016). Throughout the 10 days prior to the target pesticide application in 2020, hourly particulate matter (with diameters ≤ 2.5 µm) measured in Sacramento fluctuated from 5 to 264 μg m^−3^ and averaged 46 μg m^−3^ (station number: 060670006; US EPA 2022a). High particulate matter concentrations may have affected naled and dichlorvos degradation, resulting in the detection of dichlorvos in macroinvertebrates 10 days after application. This study was not designed to assess whether particulate matter may have intercepted airborne naled droplets because all water samples were filtered prior to extraction, which removed any particulates. Determining the effect of wildfire smoke on the persistence of pesticides and exposure to non-target organisms would allow resource managers to make informed application decisions in the changing climate.

## Conclusions

This study presents evidence that (1) concentrations of naled and dichlorvos that exceeded California National Pollutant Discharge Elimination System (NPDES) values and aquatic life toxicity benchmarks were detected in water in lentic and lotic systems the day after a spray, (2) naled and dichlorvos were transported away from the target spray areas, and (3) dichlorvos persisted in aquatic macroinvertebrates for 3–10 days following an aerial application. Naled and dichlorvos degraded quickly in the aquatic environments, as neither compound was detected in water samples collected more than 1 day after the target ultra-low volume (ULV) application. The concentrations of naled and dichlorvos in water likely were affected by ecosystem type and vector control flight paths, and there was substantial spatial variation in concentrations within an ecosystem. Detection and persistence of dichlorvos in crayfish occurred in lentic waters in this study, and there may be potential effects to higher trophic levels that consume local crayfish.

## Data Availability

The datasets generated during and/or analyzed during the current study are available in Smith et al. (2022) at https://doi.org/10.5066/P9F3DU0U.
